# Chitosan-Stabilized Selenium Nanoparticles and Metformin Synergistically Rescue Testicular Oxidative Damage and Steroidogenesis-Related Genes Dysregulation in High-Fat Diet/Streptozotocin-Induced Diabetic Rats

**DOI:** 10.3390/antiox10010017

**Published:** 2020-12-27

**Authors:** Yasmina M. Abd El-Hakim, Amany Abdel-Rahman Mohamed, Safaa I. Khater, Ahmed Hamed Arisha, Mohamed M. M. Metwally, Mohamed A. Nassan, Manal Ewaiss Hassan

**Affiliations:** 1 Department of Forensic Medicine and Toxicology, Faculty of Veterinary Medicine, Zagazig University, Zagazig 4511, Egypt; 2 Department of Biochemistry, Faculty of Veterinary Medicine, Zagazig University, Zagazig 4511, Egypt; safaa_khater83@yahoo.com; 3 Department of Physiology, Faculty of Veterinary Medicine, Zagazig University, Zagazig 44511, Egypt; vetahmedhamed@zu.edu.eg; 4 Department of Animal Physiology and Biochemistry, Faculty of Veterinary Medicine, Badr University in Cairo (BUC), Badr City, Cairo 11865, Egypt; 5 Department of Pathology, Faculty of Veterinary Medicine, Zagazig University, Zagazig 44511, Egypt; metywally@gmail.com; 6 Department of Clinical Laboratory Sciences, Turabah University College, Taif University, P.O. Box 11099, Taif 21944, Saudi Arabia; m.nassan@tu.edu.sa; 7 Department of Biochemistry and Molecular Biology, Faculty of Medicine, Beni-Suef University, Beni-Suef 62521, Egypt; manalewais11@gmail.com; 8 Department of Pathology, Medical College, Jouf University, Al-Jawf 72388, Saudi Arabia

**Keywords:** chitosan-stabilized selenium nanoparticles, oxidative stress, male fertility, testicular dysfunction, type 2 diabetes mellitus, metformin, steroidogenesis related genes

## Abstract

Background: this study examined the metformin (MF) and/or chitosan stabilized selenium nanoparticles (CH-SeNPs) efficacy to alleviate the male reproductive function impairment in a high-fat diet feed with low-dose streptozotocin (HFD/STZ) induced type 2 diabetes mellitus (T2DM) diabetic rat model. Methods: control non-diabetic, HFD/STZ diabetic, HFD/STZ+MF, HFD/STZ+CH-SeNPs, and HFD/STZ+MF+CH-SeNPs rat groups were used. After 60 days, semen evaluation, hormonal assay, enzymatic antioxidant, lipid peroxidation, testis histopathology, and the steroidogenesis-related genes mRNA expressions were assessed. Results: in the HFD/STZ diabetic rats, sperm count and motility, male sexual hormones, and testicular antioxidant enzymes were significantly reduced. However, sperm abnormalities and testicular malondialdehyde were significantly incremented. The steroidogenesis-related genes, including steroidogenic acute regulatory protein (StAr), cytochrome11A1 (CYP11A1), cytochrome17A1 (CYP17A1), and hydroxysteroid 17-beta dehydrogenase 3 (HSD17B3), and the mitochondrial biogenesis related genes, including peroxisome proliferator-activated receptor gamma coactivator 1-alpha (PGCα) and sirtuin (SIRT), were significantly downregulated in the HFD/STZ diabetic rats. However, CYP19A1mRNA expression was significantly upregulated. In contrast, MF and/or CH-SeNPs oral dosing significantly rescued the T2DM-induced sperm abnormalities, reduced sperm motility, diminished sexual hormones level, testicular oxidative damage, and steroidogenesis-related genes dysregulation. In the MF and CH-SeNP co-treated group, many of the estimated parameters differ considerably from single MF or CH-SeNPs treated groups. Conclusions: the MF and CH-SeNPs combined treatment could efficiently limit the diabetic complications largely than monotherapeutic approach and they could be considered a hopeful treatment option in the T2DM.

## 1. Introduction

Diabetes mellitus (DM) is a metabolic condition that is determined by continuous blood glucose increases due to impaired production and/or insulin action [1]. Recent information demonstrates that the world’s diabetic patient number is more than 415 million, and this number is expected to cross 642 million by 2040 [2]. Diabetes hyperglycemia is the cause of many disorders, including cardiovascular disease, neuropathy, retinopathy, and nephropathy [3].

Today, many diabetic males have been shown to have the complications of infertility [4]. Besides, the DM frequency is higher in men than in women [5]. Epidemiological studies have shown that approximately 50% of diabetic patients suffer from various levels of reproductive disorders, which include declined libido and impotence, difficulty ejaculating, erectile dysfunction, and reduced infertility [6,7,8]. Numerous studies in diabetic men and diabetic animal models showed that infertility was associated with altered spermatogenesis, degenerative testicular changes, disturbed glucose metabolism in blood testes barrier, and reduced testosterone (TES) secretion [9,10]. Oxidative stress is one of the most significant pathogenesis of sperm dysfunction that is related to DM [11]. Long-term elevated blood glucose induces an excessive reactive oxygen (ROS) production that can interrupt the oxidant-antioxidant system equilibrium [12]. Meanwhile, excessive ROS release and the resulting oxidative stress promote germ cell death and interfere with the process of spermatogenesis [13].

Type 2 diabetes mellitus (T2DM) is a heterogeneous condition that is characterized by a gradual decrease in insulin action, accompanied by beta cells’ incapacity to compensate for insulin resistance [14]. Earlier studies confirmed that the combined high-fat diet (HFD) feed with low-dose streptozotocin (STZ) treated rats serves as an alternate animal model for mimicking human syndrome, T2DM, which is also ideal for evaluating T2DM antidiabetic agents [15,16]. In the HFD/STZ diabetic model, the feeding of HFD produces insulin resistance and a low dose of STZ treatment causes the initial beta cell dysfunction that closely simulates human T2DM natural metabolic events [17].

Many drugs for DM are currently available. Metformin (MF) is the first-line drug for the management of T2DM that can effectively regulate glucose by reducing sugar absorption in the gut, increasing intracellular glucose transport, and inhibiting glycogen production in the liver [18]. In diabetes patients, MF was used to regulate blood glucose levels and avoid complications with diabetes, like diabetic cardiomyopathy [19] and retinopathy [20]. Even if MF affects other T2DM complications, testicular dysfunction treatment by MF was not reportable, and testicular tissue mechanisms remain unexplored [21].

Given the many drawbacks of DM, patients are increasingly demanding safer compounds that reduce these complications, along with antidiabetic properties [3]. At this time, increasing interest in natural supplements is geared towards using them as reproductive therapies [22,23,24,25,26]. Selenium (Se) is a crucial micronutrient in preventing and treating many disease conditions [27,28]. This metalloid micronutrient is the leader of several Se-dependent enzymes. The selenium nanoparticles (SeNPs) are known as a new Se compound with great antioxidant activity and lower toxic characteristics than other selenospecies [29,30]. Zhang et al. [31] reported that Se-NPs in mice are seven times lower in toxicity than sodium selenite and three times lower than organic Se. SeNPs demonstrated novel antioxidant properties in vivo and in vitro via the activation of selenoenzymes, which inhibits in vivo free radicals from harming tissues and cells [32]. SeNPs have also shown powerful involvement in antihyperglycemic [33].

Chitosan is a rich natural polysaccharide, the *N*-deacetylates product of chitin [34]. Chitosan is well studied because of its immense features, including biocompatibility, anti-immunogenicity, biodegradability, and safety [35]. Chitosan-based agents are extremely active nutraceuticals in diabetes prevention and treatment [36]. Chitosan was also reported to be a strong SeNP stabilizer. Moreover, Luo et al. [37] reported that selenite-loaded chitosan nanoparticles (CH-SeNPs) possessed strong antioxidant activity relative to pure selenite. 

Because several pathways are implicated in T2DM reproductive complications, combination therapy may be necessary for successful protection, rather than monotherapy. Hence, in the present study, we aimed to test the single or combined oral dosing of MF and CH-SeNPs capacity to rescue T2DM induced male reproductive dysfunction. Semen assessment, hormonal analysis, testicular antioxidants enzymes, and lipid peroxidation biomarkers were evaluated in adult male diabetic rats in order to accomplish this aim. Additionally, a histopathological assessment of testicular tissues was performed. Besides, the expression of steroidogenesis and mitochondria biogenesis related genes was assessed for exploring the underlying mechanisms

## 2. Materials and Methods

### 2.1. Tested Compounds

The STZ from Sigma–Aldrich (Sigma, St Louis, MO, USA) was obtained. MF was purchased as tablets containing 500 mg (Eva Pharma, Cairo, Egypt). The preparation and characterization of CH SeNPs have been previously described in our earlier study [38]. All other reagents/chemicals used were of analytical grade (Sigma, St. Louis, MO, USA).

### 2.2. Experimental Animals

One hundred *Sprague-Dawley* male rats (nine weeks old; 280 ± 5 g) were obtained from the laboratory animal’s farm, Faculty of Veterinary Medicine, Zagazig University. All of the rats were held in cages of stainless steel maintained in an atmosphere free of pathogens at 21–24 °C, 60% relative humidity, and a 12-h light-dark cycle. The rats received *ad libitum* filtered water and were fed a regular rodent chow. Before starting the experiment, the rats were allowed to acclimatize for two weeks.

### 2.3. Animals and Experimental Design

The rats were weighed and randomly classified into five groups (twenty rats/group), as follows:

The control non-diabetic group: the distilled water was orally given to rats using a gastric feeding needle (1 mL/rat) throughout the whole experimental duration, except at the day analog to STZ injection in the other groups. On this day, the rats in this group were intraperitoneally injected with citrate buffer, which is used for STZ preparation in the other groups. 

The HFD/STZ diabetic group: T2DM was earlier induced following the protocol of Abdulmalek and Balbaa [39]. The rats were kept on an HFD (with 4900 kcal/kg gross caloric value comprising 14.5% protein of butter and chose casein, 58% fat of corn oil and beef tallow) for eight successive weeks [40,41]. After an overnight fast, the rats were intraperitoneally injected with STZ (35 mg/kg b.wt.), which was freshly set in 0.05 M citrate buffer (pH 4.5) [42]. All rats’ blood glucose level was checked every three days by an Accu-Chek^®^ blood glucose meter (Roche Diagnostics, Basel, Switzerland) from the tail vein. The rats showed stable hyperglycemia (200 mg/dL blood glucose levels).

The HFD/STZ + MF group: in which HFD/STZ animals were given MF that was dissolved in distilled water (500 mg/kg b.wt.) [43].

The HFD/STZ + CH-SeNPs group: in which HFD/STZ animals were orally given CH-SeNPs (2 mg Se/kg b.wt.), sonicated in distilled water for dispersing nanoparticles [44].

The HFD/STZ+MF+CH-SeNPs group: in which HFD/STZ animals received both MF and CH-Se-NPs at the equivalent declared doses and routes.

All of the treatments were given orally for 60 days. Throughout the experiment, the toxicity signs and mortality have been carefully observed.

### 2.4. Sampling

At the experiment’s termination, the rats were fasted overnight, weighed, anesthetized with sodium pentobarbital (I/P., 100 mg/kg), and the blood samples from all of the rats were collected in tubes that were devoid of an anticoagulant from the medial canthus. The tubes were left for 20 min. at room temperature, centrifuged at 3000 rpm for 10 min. The collected serum was carefully extracted and kept at −20 °C to be used for biochemical analyses. The rats were then euthanized. The rat testes (one side of 15 rats/group) were dissected and immediately transferred in liquid nitrogen to be stored at −80 °C for gene expression (RT-qPCR). Other sided ones (15 sample/group) were homogenized by Ultra-Turrax homogenizer in a cold solution of 0.015 M NaPOH buffer and 0.15 M NaCl (1:6 *w*/*v*; pH 7.8) for homogenate preparation for oxidative stress assessment. Other parts were processed for histopathological examination.

### 2.5. Semen Evaluation

For the sperm collection, cauda epididymis of all rat testis was collected and then transferred to a sterilized Petri dish with 2 mL normal saline. A small opening with sterilized scissors was then made in order to assist sperms passing through the epididymis for a spermiogram examination of the epididymis’ suspension. At 400× magnification, Slott et al. [45] protocol was used to calculate the sperm motility percentage. In the meantime, the concentration of sperm cells/milliliter semen was carried out using consistently using Robb et al. [46] technique. Sperms abnormalities have been determined following the Filler [47] procedures. Five hundred sperm cells have been checked per rat in order to determine the anomalies’ occurrence in the head, neck/mid-piece, and tail.

### 2.6. Hormonal Assay

The commercial Elabscience^®^ Biotechnology Inc.’s commercial kits (cat No.: MBS282195, MBS2502190, MBS764675, and MBS263466, respectively) with the Zirkin and Chen [48] protocols were used for TES, follicle-stimulating hormone (FSH), luteinizing hormones (LH), and estradiol (E2).

### 2.7. Analysis of Oxidants/Antioxidants Status of Testicular Tissue

The tissue homogenates of testicular tissue were used in the malondialdehyde (MDA) detection following the method of Nair and Turner [49]. Superoxide dismutase (SOD) and catalase (CAT) were estimated in line with the protocols put forward by Misra and Fridovich [50] and Sinha [51], respectively. 

### 2.8. Real-Time Quantitative PCR (RT-qPCR) Analysis

Initially, the total RNAs were extracted from testis tissue using a Trizol Reagent (Thermo Fisher Scientific; Waltham, MA, USA) in line with the manufacturer’s instructions. Two-step real-time PCR was adopted for gene expression assessment [52,53]. Briefly, cDNA synthesis by a HiSenScript™ RH (-) cDNA Synthesis Kit (iNtRON Biotechnology Co., Seongnam, Korea) in a Veriti 96-well thermal cycler (Applied Biosystems, Foster City, CA, USA) next real-time PCR in a Mx3005P Real-Time PCR System (Agilent Stratagene, Santa Clara, CA, USA) via 5× HOT FIRE Pol EvaGreen qPCR Mix Plus (Solis BioDyne, Tartu, Estonia) were accomplished. Sangon Biotech (Beijing, China) synthesized all of the primers, as presented in Table 1. The real-time PCR cycling conditions involved initial denaturation for 12 min at 95 °C, denaturation for 40 cycles for 20 s at 95 °C, annealing for 30 s at 60 °C, and extension for 30 s at 72 °C. The relative level of expression of the target genes was normsalized to GAPDH, and the relative folding changes in the gene expression had been estimated while using the 2^−ΔΔCT^ approach for comparison [54].

### 2.9. Histopathological Studies

After the experiment, the right testis from ten rats/group was examined according to standardized necropsy procedures (Kittel et al., 2004). The samples were fixed in 10% neutral buffered formalin for 72 h, washed in running water for one hour, dehydrated in ascending grades of ethyl alcohol (70–100%), cleared in xylene, impregnated, and then embedded in paraffin. The paraffinized blocks were sectioned at four-microns, which were stained with hematoxylin and eosin, mounted in DPX, covered by glass covers, examined microscopically, and histological alteration was recorded [55]. For each rat, nine images (three 10×, three 40× of nearly round nonduplicated randomly selected seminiferous tubules, and three 40× of intertubular interstitial areas) were snapshotted while using AmScope digital microscope. Subsequently, these images were used for morphometric histological assessment and lesion scoring. The 10× images were used for calculating the numbers of seminiferous tubules/image, the mean diameter of three nearly round seminiferous tubules/animal (smallest diameter + longest diameter ÷ 2), the heights of the germinal epithelium of three nearly round seminiferous tubules/animal, the proportions of seminiferous tubules exhibited germ cell depletion, vacuolations, necrosis, and giant cell formation to the total number of seminiferous tubules/image, and determining the frequencies of interstitial congestion, edema, hemorrhage, and leukocyte infiltrates/image. The 40× images were used for determining the Sertoli cells, spermatogonia, spermatocytes, and spermatids numbers in three nearly round seminiferous tubules/animals and the number of Leydig cells in three intertubular interstitial area/animal. The distinction between the Sertoli cells, spermatogonial cells, spermatocytes, and spermatids was performed following the morphological criteria; nuclear location, size, shape, and chromatin pattern. All of the measurements were carried out while using AmScope ToupView V3.7.13522 software, AmScope, Irvine, CA, USA was used to perform all the measurements. The results were presented as percentages (means ± SE).

### 2.10. Data Analysis

The homogeneity test has been conducted to ensure that all of the data are normal, and Levene’s tests have been carried out to help Levene’s study of variance assumptions (ANOVA). The data were analyzed with a one-way ANOVA, then the post hoc Tukey test, which specified the significance when *p* < 0.05. The statistical analyses were carried out while using software Prism 7.0 GraphPad (Graph-Pad, San Diego, CA, USA).

## 3. Results

### 3.1. Effect of MF and/or CH-SeNPs on Spermiogram

**Figure 1:**
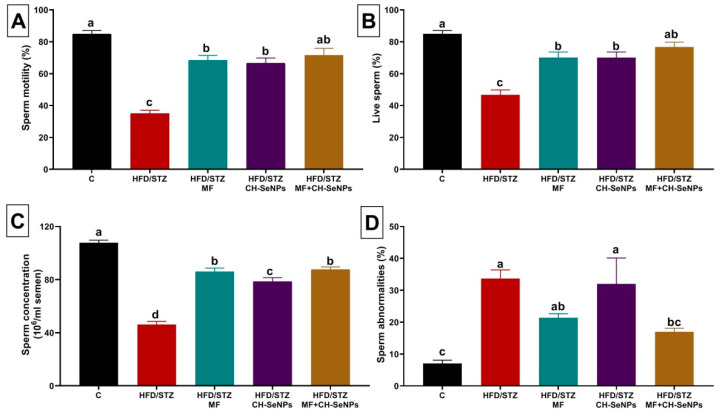
Effect of metformin (MF) and/or chitosan stabilized nanoparticles (CH-SeNPs) oral dosing for 60 days on sperm characteristics including (**A**) sperm motility, (**B**) live sperms, (**C**) sperm concentration, and (**D**) sperm abnormalities in high-fat diet/streptozotocin (HFD/STZ) diabetic male rats. Data expressed as mean ± SE, *n* = 15 for each group. Each bar carrying different letters (a–d) was significantly different at *p* < 0.05.

The HFD/STZ diabetic group displayed a significant decline (*p* < 0.001) in sperm motility percentage, sperm concentration, and live sperm percentage by −58.82%, 45.10%, and 57.28%, respectively, when compared to the control group, as presented in Figure 1. However, the sperm abnormalities percent was significantly (*p* = 0.001) incremented by approximately fourfold in the HFD/STZ diabetic rats relative to the control group. However, MF and/or CH-SeNPs oral dosing significantly restored the sperm count, motility, and live sperm percentage, but reduced sperm abnormalities percent as compared to the HFD/STZ diabetic group. Noteworthy, among all the groups, only the combined HFD/STZ+MF+CH-SeNPs group efficiently reduced the sperm abnormalities until they became not significantly varied from the control groups.

### 3.2. Effect of MF and/or CH-SeNPs on Male Reproductive Hormones

**Figure 2:**
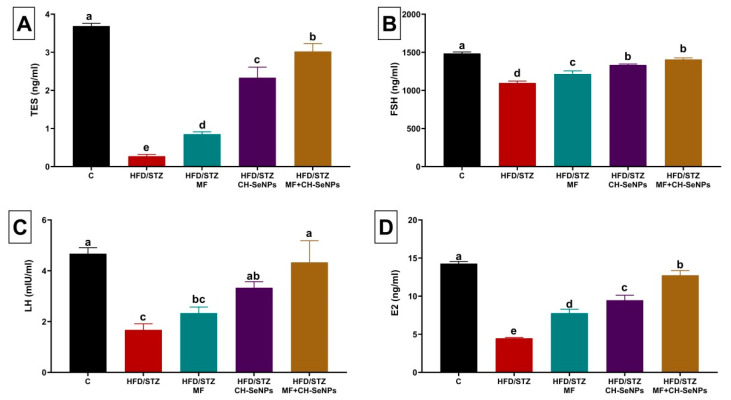
Effect of metformin (MF) and/or chitosan stabilized nanoparticles (CH-SeNPs) oral dosing for 60 days on sexual hormonal variables including (**A**) testosterone (TES), (**B**) follicle-stimulating hormone (FSH) (**C**), luteinizing hormone (LH), and (**D**) estradiol (E2) levels in the serum of HFD/STZ diabetic male rats. Data are expressed as mean ± SE, *n* = 15 for each group. Each bar carrying different letters (a–e) was significantly different at *p* < 0.05.

The HFD/STZ diabetic group exhibited a significant (*p* < 0.001) drop in the TES, FSH, LH, and E2 levels by 92.68%, 26.13%, 64.29%, and 68.54%, respectively, relative to the control group, as displayed in Figure 2. Nonetheless, MF and/or CH-SeNPs dosing to the diabetic rats significantly (*p* < 0.001) reestablished the sexual hormone levels when compared to the HFD/STZ diabetic group. The joint HFD/STZ+MF+CH-SeNPs group competently restored the TES, LH, and E2 levels until it became not significantly changed from the control group.

### 3.3. Effect of MF and/or CH-SeNPs on Testicular Antioxidants and Lipid Peroxidation Level

**Figure 3:**
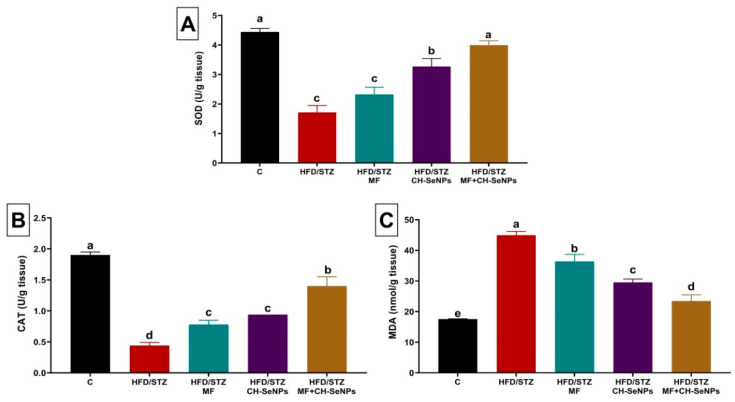
Effect of metformin (MF) and/or chitosan stabilized nanoparticles (CH-SeNPs) oral dosing for 60 days on (**A**) superoxide dismutase (SOD) (**B**), catalase (CAT), and (**C**) malondialdehyde (MDA) levels in the testicular tissues of HFD/STZ diabetic male rats. Data are expressed as mean ± SE, *n* = 15 for each group. Each bar carrying different letters (a–e) was significantly different at *p* < 0.05.

The HFD/STZ diabetic rats testicular tissues showed a significant (*p* < 0.001) depletion of the enzymatic antioxidants (SOD and CAT) by 61.54% and 76.86%, respectively, relative to the control group (Figure 3). Nevertheless, a significant (*p* < 0.001) rise (twofold) of the MDA amount was evident. However, the SOD and CAT levels were significantly (*p* < 0.001) restored in the testicular tissue of MF and/or CH-SeNPs administered diabetic rats relative to the HFD/STZ diabetic group. Moreover, in all MF and/or CH-SeNPs treated diabetic group, the MDA elevation in testicular tissue was significantly (*p* < 0.001) repressed as compared to the HFD/STZ diabetic group. Notably, the combined MF and CH-SeNPs co-treatment showed significant improvement in the SOD, CAT, and MDA levels than the single exposure to each treatment in diabetic rats.

### 3.4. Effect of MF and/or CH-SeNPs on Gene Expression in Testicular Tissue

**Figure 4:**
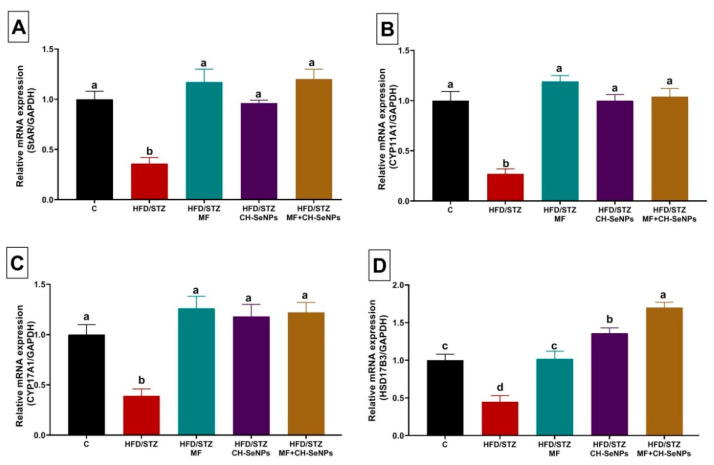
Effect of metformin (MF) and/or chitosan stabilized nanoparticles (CH-SeNPs) oral dosing for 60 days on mRNA expression of (**A**) steroidogenic acute regulatory protein (StAr), (**B**) cytochrome11A1 (CYP11A1), (**C**) cytochrome17A1 (CYP17A1) and (**D**) hydroxysteroid 17-beta dehydrogenase 3 (HSD17B3) in the testicular tissues of HFD/STZ diabetic male rats. Data are expressed as mean ± SE, *n* = 15 for each group. Each bar carrying different letters (a–d) was significantly different at *p* < 0.05.

**Figure 5:**
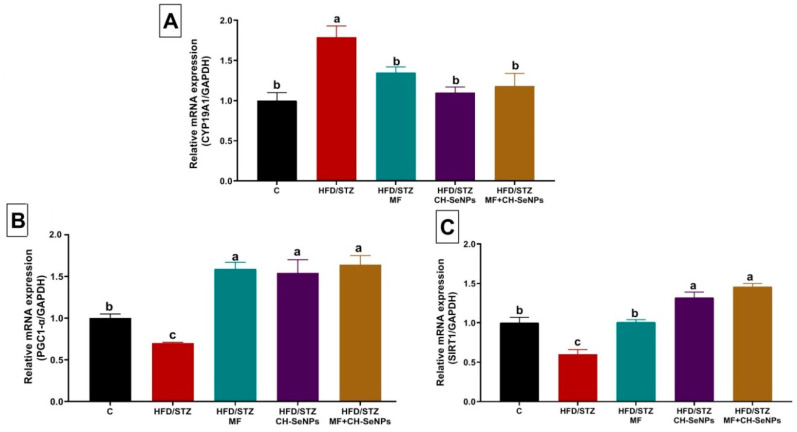
Effect of metformin (MF) and/or chitosan stabilized nanoparticles (CH-SeNPs) oral dosing for 60 days on mRNA expression of (**A**) aromatase gene (CYP19A1), (**B**) peroxisome proliferator-activated receptor gamma coactivator 1-alpha (PGC-1α), and (**C**) Silent information regulator type-1 (SIRT1) in the testicular tissues of HFD/STZ diabetic male rats. Data expressed as mean ± SE, *n* = 15 for each group. Each bar carrying different letters (a–d) was significantly different at *p* < 0.05.

Steroidogenesis-related genes, including steroidogenic acute regulatory protein (StAr), cytochrome11A1 (CYP11A1), cytochrome17A1 (CYP17A1), and hydroxysteroid 17-beta dehydrogenase 3 (HSD17B3) were significantly (*p* < 0.001) downregulated (0.36 ± 0.06, 0.27 ± 0.05, 0.39 ± 0.07, and 0.45 ± 0.08, respectively) in testicular tissue of HFD/STZ diabetic rats as compared with the control group (1.00 ± 0.08, 1.00 ± 0.09, 1.00 ± 0.10, and 1.00 ± 0.08, respectively) (Figure 4). Additionally, the mitochondrial biogenesis related genes, including Peroxisome proliferator-activated receptor gamma coactivator 1-alpha (PGCα) and Sirtuin (SIRT). were significantly (*p* < 0.001) downregulated (0.70 ± 0.01, and 0.60 ± 0.06, respectively) in testicular tissue of HFD/STZ diabetic rats when compared with the control group (1.00 ± 0.05 and 1.00 ± 0.07, respectively) (Figure 5). However, the mRNA expression levels of CYP19A1 was significantly (*p* = 0.002) upregulated in testicular tissue of HFD/STZ diabetic rats (1.79 ± 0.14) as compared with the control group (1.00 ± 0.10) (Figure 5).

The MF and/or CH-SeNPs dosing to the diabetic rats significantly (*p* < 0.001) reversed the dysregulation of the analyzed steroidogenesis and mitochondrial biogenesis related genes when compared with the HFD/STZ diabetic rats. Notably, the combined MF and CH-SeNPs co-treatment showed a significant improvement in the mRNA expression levels of HSD17B3 than the single exposure to each treatment in diabetic rats.

### 3.5. Histopathological Findings

Grossly, the testes of the examined control rats displayed no pathological alterations and were normal. Still, those of HFD/STZ and HFD/STZ+CH-SeNPs -treated rats showed a minor decrease in the sizes and weights when compared to the control, besides the vascular congestion of the *tunica vaginalis*. Testes from HFD/STZ+MF and HFD/STZ+MF+CH-SeNPs treated rats were normal, except for mild congestion of the *tunica vaginalis*.

**Figure 6:**
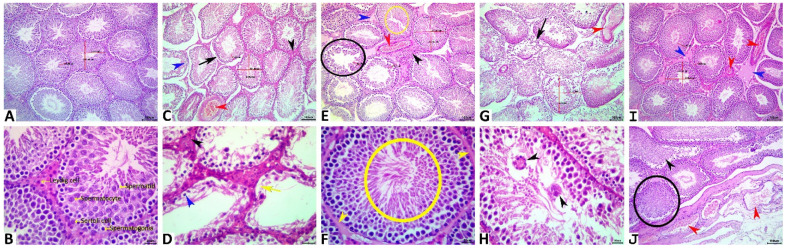
Representative photomicrograph of H&E stained testicular tissue sections showing normal histological picture in the control rats (**A**,**B**). HFT/STZ testes showing congestion (red arrowhead), vacuolated germinal epithelium (blue arrowhead), necrotic germinal epithelium, (yellow arrow), interstitial fibrosis (black arrowhead), and redundant basal lamina (arrow) (**C**,**D**). HFT/STZ+MF testes showing germ cell depletion (black ellipse), spermatid retention (yellow ellipse), thickened basal lamina (yellow arrowhead), interstitial edema (blue arrowhead), fibrosis (black arrowhead), and congestion (red arrowhead) (**E**,**F**). HFT/STZ+CH-SeNPs testes showing congestion (red arrowhead), multinucleated giant cell s (black arrowheads) and redundant basal lamina (arrow) (**G**,**H**). HFT/STZ+MF+CH-SeNPs testes showing congestions (red arrowheads), desquamated germinal epithelium (black arrowhead), and sperm stasis (ellipse) (**I**,**J**). Scale bar is 100 microns for (**A**,**C**,**E**,**G**,**I**), and 20 microns for (**B**,**D**,**F**,**H**,**J**).

Microscopically, the control rats testes displayed normal histological architectures [smoothly rounded seminiferous tubules (14.20 ± 0.36/10× image with mean diameter, 255.36 ± 1.37 um, and germinal cell height, 83.22 ± 1.70 um) that were lined by pyramidal Sertoli cells (30.50 ± 0.48/ST) sustaining successive populations of maturing germ cells; spermatogonia (66.90 ± 1.16), spermatocytes (147.30 ± 1.46), and spermatids (221.30 ± 5.14), and peritubular connective tissue containing Leydig cells (13.60 ± 0.45/intertubular area), fibroblasts, myoid cells, and blood vessels] (Figure 6A,B). The testes from the HFD-STZ-treated rats exhibited a vast array of histopathological alteration, including degenerative changes [increased numbers of STs/10× image (18.20 ± 0.33), due to decreased mean diameters (224.74 ± 4.67 um), tubular thinning due to diminished heights of germinal epithelium/ST (58.09 ± 1.94 um), germ cell depletion, vacuolation, disorganization, and desquamation, and basal lamina with irregular thickness, or lacking the smoothly rounded profile, or broken and or redundant], necrotic changes [few tubules showed focal or entire loss of the germinal epithelium, besides the decreased numbers of Sertoli cells (26.20 ± 0.88/ST) and interstitial Leydig cells (10.40 ± 0.45/interstitial area)], vascular and inflammatory changes (most specimens showed interstitial congestion, and edema but few showed interstitial mononuclear cell infiltrate and/or minute hemorrhage), and progressive changes (interstitial fibroblastic proliferation and intratubular spermatid giant cell formation) (Figure 6C,D). A significant reduction in the frequencies and severities of the diabetes-induced orchiopathy was seen in the testes of HFD/STZ+MF-treated rats. Yet, some histopathological alterations, including germ cell depletion, spermatid retention, thickened basal lamina, interstitial congestion, and edema with few mononuclear cell infiltrations, were evident (Figure 6E,F). The testes of HFD/STZ +CH-SeNPs -treated rats showed the same histopathological alterations as those detected in the HFD/STZ-treated rats. Still, the inflammatory, vascular, and proliferative interstitial tissue changes were minimal (Figure 6G,H).

Interestingly, the testes of HFD/STZ+MF+CH-SeNps -treated rats showed a sharp decline in the diabetes-induced orchiopathy, but the testes did not maintain their normal histology. A few histopathological alterations were still observed, including sperm stasis and germ cell desquamation of few seminiferous tubules and interstitial edema and congestion of the interstitial tissue and *tunica vaginalis* (Figure 6G,H). Table 2 summarizes the morphological evaluation and the quantitative lesion scoring in all groups.

## 4. Discussion

Initially, in our earlier work [38], the HFD/STZ treated rats showed clear hyperglycemic condition (276.74 ± 6.03 mg blood glucose/dl) and hyperinsulinemia (36.84 ± 0.58 mg insulin/dL) when compared to the control group (95.72 ± 0.42 and 11.62 ± 0.25, respectively). The hyperglycemia and insulin resistance could be related to the STZ induced selective pancreatic β-cells damage, which mainly linked to DNA alkylation and, to a lesser extent, ROS generation, and nitric oxide [17]. However, in our previous study [38], the MF and/or CH-SeNPs oral dosing, particularly the combined therapy, significantly suppressed the HFD/STZ induced increment in the glucose and insulin levels. Thus, the earlier study proposed the potent antidiabetic activity of CH-SeNPs and MF combined therapy, which could be related to the CH-SeNPs capacity to renew the beta cells activity, decreasing the blood glucose level and enhancing insulin release [56]. Se can also induce mimetic-insulin effects by activating Akt and other kinases that are responsible for insulin signaling, such as p70 S6 kinase [57].

Herein, the diabetic HFD/STZ rats displayed a pronounced decrease in the functional characteristics of the sperm, as demonstrated by a decrease in sperm count, motility, and viability with a rapid rise in morphological abnormalities. Besides, in our diabetic rat model system, a significant decline in the germinal epithelium disintegration and germ cell reduction was observed. There is evidence that normal lipid metabolism is crucial for normal spermatogenesis. Hyperglycemia is often associated with abnormalities in lipid profiles, like elevated serum levels of total cholesterol, triglycerides, low-density lipoprotein cholesterol, and declined serum levels of high-density lipoprotein cholesterol [38,58]. Increased ROS in diabetes also has a major impact on sperm, because of the presence in the cell membrane of various polyunsaturated fatty acids [59]. In recent years, increasing evidence has shown that ROS release with hyperglycemia can interrupt the blood-testis barrier and worsen sperm dysfunction [60,61]. The negative effects of T2DM on sperm development and characteristics in the present study comply with previous studies [62,63]. In this context, the decreased volume and quality of sperm are associated with infertility [64].

The MF and/or CH-SeNPs oral dosing significantly restored the sperm count, motility, and live sperm percentage concomitantly with reduced sperm abnormalities percent. The potent antihyperglycemic of MF could reduce ROS release and maintain sperm integrity and viability [65]. Besides, Zhou et al. [66] described the AMP-activated protein kinase (AMPK) by MF. Several shreds of evidence suggest that AMPK is present in spermatozoa and it plays vital roles in spermatozoa motility, the spermatozoa membrane’s quality, and antioxidant molecules [67,68,69]. CH-seNPs are beneficial to the quality of semen, mainly because of their antioxidant ability and their capacity to prevent lipid peroxidative damage by free radicals scavenging [29]. In the same respect, Liu et al. [70] found that 0.2 mg/kg bwt of oral gavage-administered SeNPs in male Sprague-Dawley rats for two weeks increased sperm concentration, vitality, and movement indicators.

Our data also revealed that the induction of T2DM significantly reduced the TES, FSH, LH, and E2 levels in the male rats. Earlier studies in brain-specific insulin receptor knockout mice confirmed the link between fertility and brain insulin signaling [71]. This expected mechanism could be, as follows: inadequate insulin brain signaling in the diabetic patients elicits a negative effect on the pituitary function, reduces the hormonal output, reduces the LH effect on the Leydig cell to produce TES, and upsets FSH action on the Sertoli cell in order to produce sperm [71]. In this regard, up to 1/3 of men with T2DM have substantially sub-normal total and free TES levels [72]. In male mice, with nicotinamide/STZ-induced T2DM serum TES, LH levels, sperm count, and viability were also significantly decreased [73].

Interestingly, the TES, FSH, LH, and E2 levels re-establishment after MF and/or CH-SeNPs treatment showed their protective impact against reproductive hormonal insufficiencies resulted from T2DM in the rat model. Nasrolahi et al. [74] similarly conducted a trial of diabetic male rats that were induced by STZ and indicated that treatment with MF increased serum TES. MF has also shown its effectiveness in managing Leydig cell steroidogenesis and enhancing TES [75]. On the other hand, SeNPs restored the depleted sexual male hormone levels due to deltamethrin exposure in rats [76].

Oxidative stress is one of the main underlying mechanisms in diabetic sperm dysfunction [77]. Thus, in order to identify MF and/or CH-SeNPs, protective mechanisms against T2DM mediated reproductive toxicity, oxidative stress indices, essential antioxidant enzymes, and lipid peroxidation in testicular homogenate have been assessed. Initially, the diabetic rats displayed an obvious decline in the antioxidant enzyme activities (CAT and SOD) simultaneously with a sharp rise in MDA content. T2DM induced hyperglycemia can lead to higher tissue oxidative stress and further increases the disequilibrium between ROS output and enzymatic antioxidants [78]. Besides, Chodari et al. [79] demonstrated that increased oxidative stress may be attributed to TES deficiency in diabetic conditions. In the testis and sperm in the diabetes state, oxidative stress may be because of a hypoxic state, since diabetes induces a glycosylated formation of hemoglobin that impedes the delivery of oxygen on the testes [80].

In contrast, MF and/or CH-SeNPs oral dosing significantly restored the depleted antioxidant enzymes and suppressed the elevated MDA content in testicular tissue of diabetic rats. It was stated that standard medicinal product MF exhibited antioxidant properties in mitochondrial breathing, thereby increasing the antioxidant enzymes and decreasing ROS in diabetic rats [81]. The addition of Se-NPs alone or chitosan-coated to the diet of layered chicks has recently been shown to increase total SOD, GPx, and CAT activities in erythrocytes than dietary Se [43,82]. This may be due to SeNPs’ greater antioxidant activity [29,30].

Of note, in our earlier work [38], throughout the eight week of receiving HFD, all of the rats showed the highest weight gain when compared to those that received a normal diet. Subsequently, after STZ injection, a significant reduction in the body weight was recorded in HFD/STZ treated rats when compared to the control one at the end of the experiment. In this regard, Rossmeisl et al. [83] reported that, during insulin resistance, glucose metabolism is extremely reduced and fat metabolism increases, which results in weight loss in diabetic rats. However, CH-SeNPs and/or MET oral dosing in the earlier experiment significantly restored body weight gain, being possibly linked to their antidiabetic activity.

Accumulating evidence has confirmed that testicular steroidogenesis and spermatogenesis dysfunction correlate with male reproductive failure, due to hyperglycemic oxidative stress and insulin deficiency [84]. Testicular steroidogenesis is a highly regulated cholesterol-controlled signaling pathway that is dependent on cholesterol that is accessible within testicular mitochondria controlled by StAR [85]. CYP11A1 converts the cholesterol molecule into pregnenolon in the inner mitochondrial membrane [86]. CYP17A1 catalyzes dehydroepiandrosterone synthesis from pregnenolone and the further formation of androstenedione [87]. Finally, TES formation from androstenedione and the respective back-reaction is catalyzed by HSD17B3 [88]. Importantly, aromatase (CYP19A1) catalyzes TES’s irreversible conversion and into E2 [89].

Hyperglycemia in testicular tissue has been reported to reduce the antioxidant capability in mitochondria [90]. Several transcriptional coactivators regulate mitochondrial biological activities. For instance, the peroxisome proliferator-activated receptor-gamma coactivator 1-alpha (PGC-1α) plays a chief role in controlling mitochondrial function and biogenesis. Inactivated PGC-1α is deacetylated to the active form by silent information regulator type-1 (SIRT1) [91]. Notably, spermatogenic cells’ mitochondria have many vital roles in spermatogenesis events [92].

In order to explore the protective effect of the MF and/or CH-SeNPs on the male diabetic rat’s fertility, we studied the level of expression of four key steroidogenic genes StAR, Cyp11A1, Cyp17A1, and HSD-17 B3 in testis. Initially, the testicular StAR, Cyp11A1, Cyp17A1, and HSD-17 B3 mRNA expression was suppressed, concomitant with upregulated CYP19A1 in diabetic rats, demonstrating compromised testicular steroidogenesis. Additionally, the notable downregulation of PGC-1α and SIRT1 indicates the spermatogenic mitochondrial compromise by ROS due to T2DM induced hyperglycemia. In contrast, a substantial recovery was recorded in the gene expression of steroidogenic and related genes spermatogenic mitochondria following MF and/or CH-SeNPs oral dosing.

Notwithstanding, the current data elucidated that combined treatment with MF and CH-SeNPs showed a significant improvement in TES, E2, LH, SOD, and MDA levels than the single exposure to each treatment in diabetic rats. Additionally, other estimated parameters, including sperm motility, live sperm%, sperm abnormalities, LH, and SOD were brought back to the normal control level in the HFD/STZ-MF+CH-SeNPs group. Such a synergistic effect could be linked to the resultant superior free-radical quenching capability. MF showed close synergistic interaction with other drugs, like rapamycin, in the rat model of testicular torsion/detorsion-induced ischemia/reperfusion [93]. Additionally, MF and the *Nigella sativa* dietary supplement synergistically improved serum testosterone in diabetic rats [94].

## 5. Conclusions

In conclusion, our findings provide new insights into the steroidogenesis’s potential participation (StAr, CYP11A1, CYP17A1, HSD17B3, and CYP19A1) and mitochondrial biogenesis (PGCα and SIRT) related genes in the induction of testicular damage in T2DM. Our results suggested that the MF and/or CH-SeNPs administration could be useful in the guard against T2DM accompanying male reproductive disorders by the augmentation of the antioxidant capability and the proper regulation of steroidogenesis and mitochondrial biogenesis related genes. Notably, this study showed that MF and CH-SeNPs combination have significant synergistic effects that could open up further opportunities for the design of new combinatorial remedies to the associated infertility in diabetic patients.

## Figures and Tables

**Table 1:** Primer sequences, accession number, and product size for the quantitative RT-PCR for the analyzed genes in the testicular tissue.

Gene	Forward Primer (5′–3′)	Reverse Primer (5′–3′)	Accession No	Product Size
StAr	CCCAAATGTCAAGGAAATCA	AGGCATCTCCCCAAAGTG	NM_031558.3	187
CYP11A1	AAGTATCCGTGATGTGGG	TCATACAGTGTCGCCTTTTCT	NM_017286.3	127
CYP17A1	TGGCTTTCCTGGTGCACAATC	TGAAAGTTGGTGTTCGGCTGAAG	NM_012753.2	90
HSD17B3	AGTGTGTGAGGTTCTCCCGGTACCT	TACAACATTGAGTCCATGTCTGGCCAG	NM_054007.1	161
CYP19A1	GCTGAGAGACGTGGAGACCTG	CTCTGTCACCAACAACAGTGTGG	NM_017085.2	178
PGC1-α	ATGTGTCGCCTTCTTGCTCT	ATCTACTGCCTGGGGACCTT	NM_031347.1	180
SIRT1	GGCACCGATCCTCGAACAAT	CGCTTTGGTGGTTCTGAAAGG	NM_001372090.1	119
GAPDH	GGCACAGTCAAGGCTGAGAATG	ATGGTGGTGAAGACGCCAGTA	NM_017008.4	143

StAr: Steroidogenic Acute Regulatory Protein; CYP11A1: Cytochrome P450 Family 11 Subfamily A; CYP17A1: Cytochrome P450 Family 17 Subfamily A; HSD17B3: 17-beta hydroxysteroid dehydrogenase 3 Member 1; CYP19A1: Cytochrome P450 Family 19 Subfamily A; PGC-1α: Peroxisome proliferator-activated receptor gamma coactivator 1-alpha; SIRT1: Sirtuin 1; GAPDH: Glyceraldehyde-3-Phosphate Dehydrogenase.

**Table 2:** Effect of metformin (500 mg/kg bwt) and/or chitosan-stabilized selenium nanoparticles (2 mg Se/kg bwt) on lesion scoring of testicular tissues of HFD/STZ-diabetic rats.

Lesion	Control	HFD/STZ	HFD/STZ+MF	HFD/STZ+CH-SeNPs	HFD/STZ+MF+CH-SeNps
Spermatogonial cells/ST	66.90 ^a^± 1.16	48.50 ^c^ ± 0.96	59.80 ^b^ ± 1.16	49.90 ^c^ ± 0.92	62.50 ^b^ ± 0.86
Spermatocytes/ST	147.30 ^a^ ± 1.46	105.10 ^d^ ± 1.37	128.30 ^c^ ± 3.25	111.50 ^e^ ± 1.42	140.70 ^b^ ± 1.80
Spermatid/ST	221.30 ^a^ ± 5.14	170.40 ^c^ ± 2.97	199.30 ^b^ ± 1.04	174.70 ^c^ ± 2.41	212.60 ^a^ ± 3.69
Sertoli cells/ST	30.50 ^a^ ± 0.48	26.20 ^b^ ± 0.88	29.30 ^a^ ± 0.70	26.60 ^b^ ± 0.78	29.80 ^a^ ± 0.59
Leydig cells/intertubular area	13.60 ^a^ ± 0.45	10.40 ^b^ ± 0.45	12.30 ^a^ ± 0.52	10.80 ^b^ ± 0.39	12.60 ^a^ ± 0.43
Height of germinal epithelium	83.22 ^a^ ± 1.70	58.09 ^c^ ± 1.94	77.04 ^b^ ± 2.50	55.55 ^c^ ± 1.83	80.16 ^ab^ ± 2.02
Numbers of STs/10X	14.20 ^b^ ± 0.36	18.20 ^a^ ± 0.33	14.80 ^b^ ± 0.44	17.20 ^a^ ± 0.47	14.50 ^b^ ±0.22
Mean diameter of ST	255.36 ^a^ ± 1.37	224.74 ^b^ ± 4.67	248.23 ^a^ ± 1.63	229.27 ^b^ ± 1.87	252.93 ^a^ ± 1.06
STs with vacuolated germinal epithelium	0.00 ^d^ ± 0.00	7.38 ^a^ ± 0.32	4.04 ^c^ ± 0.23	5.57 ^b^ ± 0.33	3.55 ^c^ ± 0.28
STs with desquamated germinal epithelium	0.00 ^d^ ± 0.00	20.10 ^a^ ± 1.32	11.14 ^b^ ± 1.23	17.10 ^a^ ± 1.77	3.74 ^c^ ± 0.42
STs with depleted germ cells	0.00 ^e^ ± 0.00	15.88 ^a^ ±1.34	7.36 ^c^ ± 0.23	11.91 ^b^ ± 0.75	3.58 ^d^ ± 0.33
STs with necrotic and or complete loss of germinal epithelium	0.00 ^e^ ± 0.00	4.83 ^a^ ± 0.43	2.05 ^c^ ± 0.17	3.53 ^b^ ± 0.22	1.10 ^d^ ± 0.10
STs with multinucleated giant cell formation	0.00 ^b^ ± 0.00	3.07 ^a^ ± 1.19	1.00 ^b^ ± 0.12	1.31 ^b^ ± 0.07	0.40 ^b^ ± 0.17
STs with spermatid retention	0.00 ^c^ ± 0.00	0.86 ^ab^ ± 0.31	0.75 ^abc^ ± 0.33	1.09 ^a^ ± 0.31	0.25 ^bc^ ± 0.17
STs with uneven, or redundant or broken basal lamina	0.00 ^e^ ± 0.00	14.47 ^a^ ± 1.01	7.40 ^c^ ±0.43	10.94 ^b^ ± 1.00	3.06 ^d^ ± 0.45
Interstitial leukocytic infiltration	0.00 ^b^ ± 0.00	10.66 ^a^ ± 1.71	3.00 ^b^ ± 0.92	9.00 ^a^ ± 1.58	1.00 ^b^ ± 0.51
Interstitial edema	0.00 ^b^ ±0.00	12.67 ^a^ ± 1.47	2.33 ^b^ ± 1.00	10.67 ^a^ ± 2.42	3.33 ^b^ ± 1.99
Interstitial congestion	0.00 ^d^ ± 0.00	15.33 ^a^ ± 1.87	4.33 ^c^ ± 0.71	9.97 ^b^ ± 1.78	2.66 ^c d^ ± 0.83
Interstitial hemorrhage	0.00 ^b^ ± 0.00	2.33 ^a^ ± 0.71	1.00 ^b^ ± 0.51	0.67 ^b^ ± 0.44	0.33 ^b^ ± 0.33

Values are mean ± SE. Means within the same row carrying different superscripts (^a–e^) are significantly different at *p* < 0.05.

## Data Availability

All datasets generated for this study are included in the article.
